# Non-conventional histologies of prostate cancer: from morphology to biology-driven precision oncology

**DOI:** 10.1093/oncolo/oyag391

**Published:** 2026-10-05

**Authors:** Fabiano Flauto, Giuseppe Neola, Alessia Cimadamore, Michele Maffezzoli, Akash Maniam, Sarah Scagliarini, Fabio Calabrò, Luigi Formisano, Pasquale Rescigno, Giuseppe Luigi Banna

**Affiliations:** Department of Clinical Medicine and Surgery, Federico II University, Naples, 80131, Italy; Department of Clinical Medicine and Surgery, Federico II University, Naples, 80131, Italy; Portsmouth Hospitals University NHS Trust, Portsmouth, PO6 3LY, United Kingdom; Department of Medicine, Institute of Pathological Anatomy, University of Udine, Udine, 33100, Italy; Department of Medicine and Surgery, University Hospital of Parma-Medical Oncology Unit, University of Parma, Parma, 43126, Italy; Portsmouth Hospitals University NHS Trust, Portsmouth, PO6 3LY, United Kingdom; UOC di Oncologia, Azienda Ospedaliera di Rilievo Nazionale Cardarelli di Napoli, Naples, 80131, Italy; Medical Oncology 1, IRCCS Regina Elena National Cancer Institute, Rome, 00128, Italy; Department of Clinical Medicine and Surgery, Federico II University, Naples, 80131, Italy; Translational & Clinical Research Institute, Centre for Cancer, Newcastle University, Newcastle Upon Tyne, NE2 4HH, United Kingdom; Portsmouth Hospitals University NHS Trust, Portsmouth, PO6 3LY, United Kingdom

**Keywords:** prostate cancer, neuroendocrine prostate cancer, aggressive-variant prostate cancer, intraductal carcinoma, non-conventional histology, lineage plasticity, DLL3, molecular pathology, precision oncology

## Abstract

Non-conventional prostate cancer histologies represent a small but clinically significant subset characterized by aggressive behavior, atypical biomarker profiles, and reduced dependence on androgen receptor signaling. Evidence indicates these histologies reflect dynamic biological states driven by genomic instability, lineage plasticity, and therapeutic selection rather than static morphologic entities, with several morphologies acting as accessible, if imperfect, surrogates for actionable alterations. We performed a translational narrative review integrating anatomic pathology, genomic and epigenomic biology, and biomarker-selected therapy, searching PubMed/MEDLINE, Embase, Cochrane CENTRAL, the ASCO Meeting Library, ESMO publications, and APCCC through May 2026, spanning neuroendocrine, ductal, intraductal, cribriform, adenosquamous, sarcomatoid, adenoid cystic, signet ring cell, and mucinous subtypes. These histologies are individually uncommon but are consistently associated with advanced stage and inferior survival. Neuroendocrine prostate cancer is the most aggressive phenotype and a major mechanism of resistance to AR-directed therapy, driven by combined RB1 and TP53 loss, epigenetic reprogramming, and lineage plasticity, and marked by DLL3 expression. Cribriform and intraductal carcinoma are often enriched for BRCA2 and DNA-repair deficiency, whereas ductal differentiation is associated with mismatch-repair deficiency in a subset, with implications for PARP inhibition and checkpoint blockade. Emerging strategies include platinum-based chemoimmunotherapy and investigational lineage-directed agents targeting DLL3, STEAP1, KLK2, and TROP-2, alongside radioligands. These histologies are best viewed as biologically distinct, evolving disease states. By integrating pathology, molecular biology, and biomarker-selected therapy into one translational pathway, this review reframes non-conventional histology as a prompt for early molecular characterization and adaptive, biology-driven treatment.

Implications for practice Non-conventional prostate cancer histologies frequently reflect aggressive, AR-indifferent disease with poor outcomes and resistance to standard therapy. Clinical features such as rapid progression, visceral metastases, or PSA disproportionately low for tumor burden should prompt metastatic biopsy and molecular reassessment. Neuroendocrine differentiation is associated with DLL3 expression and combined RB1/TP53 loss; cribriform and intraductal carcinoma with BRCA2 and DNA-repair deficiency; and ductal differentiation with mismatch-repair deficiency. These are probabilistic associations that should trigger somatic testing which, not morphology alone, guides rational use of platinum chemotherapy, PARP inhibitors, immunotherapy, and emerging lineage-directed therapies.

## Introduction

Prostate cancer is overwhelmingly acinar adenocarcinoma, but a small, clinically important subset shows non-conventional histologies with distinct morphology, behavior, and therapeutic sensitivity. The 2022 World Health Organization (WHO) classification^1^ defines these as entities separate from usual acinar adenocarcinoma and distinguishes them from adverse architectural patterns such as intraductal carcinoma of the prostate (IDC-P) and invasive cribriform growth, that arise within otherwise conventional tumors. Although individually uncommon, these histologies are disproportionately associated with advanced stage, atypical prostate-specific antigen (PSA) kinetics, and reduced androgen receptor (AR) dependence.^2^^,^^3^

Morphology, though indispensable for diagnosis, does not fully capture this heterogeneity. Contemporary genomic, epigenomic, and transcriptomic data indicate that many non-conventional morphologies are dynamic states shaped by clonal evolution, lineage plasticity, and therapeutic pressure rather than fixed categories.^4^ Crucially, several act as accessible, albeit imperfect, surrogates of actionable molecular alterations, with neuroendocrine differentiation being associated with DLL3 expression and combined RB1 and TP53 loss; cribriform and intraductal architecture for BRCA2 and broader DNA damage repair (DDR) deficiency; and ductal differentiation for mismatch repair (MMR) defects. Each association points to a specific therapeutic class and to distinct germline implications.

This principle is exemplified by neuroendocrine prostate cancer (NEPC), arising as a rare de novo phenotype or, more often, as a treatment-emergent state after sustained AR-pathway inhibition,^4^ and reinforced by ductal adenocarcinoma, IDC-P, and cribriform architecture as correlates of DDR-enriched, genomically unstable disease.^5^ Not all aggressive phenotypes are formal variants; aggressive-variant prostate cancer (AVPC) and lineage-plastic states are included here because they overlap with rare histologies and carry direct implications for testing and treatment.

Histology should therefore be read not only as description but also as an accessible surrogate of tumor biology. In this review, we integrate pathology, immunohistochemistry, molecular biology, and biomarker-informed therapy across the full spectrum of non-conventional histologies and adverse architectural patterns.

## Materials and methods

This narrative review drew on a structured search of PubMed/MEDLINE, Embase, Cochrane CENTRAL, and major oncology congresses (ASCO, ASCO GU, ESMO, and APCCC) through May 2026, combining prostate cancer terms with histology-specific and molecular descriptors (neuroendocrine, small cell, ductal, intraductal, cribriform, sarcomatoid, adenosquamous, signet ring, mucinous, adenoid cystic, aggressive variant; molecular, genomic, DLL3, BRCA2, mismatch repair, lineage plasticity, treatment). Priority was given to prospective studies, large retrospective cohorts, translational work, and recent trials; given the rarity of these entities, selected case series and reports were included, and congress abstracts are flagged as preliminary. Eligible sources were English-language and peer-reviewed or pivotal congress abstracts. No PRISMA-based selection was applied; preclinical data were used only to explain mechanism, while clinical recommendations were anchored to evidence graded by study design. The search covered records from database inception through May 2026. Title/abstract screening and study selection were performed independently by 2 authors (F.F. and G.N.), with disagreements resolved by a third author (G.L.B.). Evidence was informally graded by study design, in descending order of priority: randomized or prospective trials; large retrospective cohorts and registries; small retrospective series and case reports; and preclinical or mechanistic data, used only to explain biological rationale.

## Epidemiology and clinical outcomes

Non-acinar morphologies are individually uncommon, with estimates varying by definition (pure vs mixed) and registry; several pure subtypes each account for approximately 0.3%-0.4% of cases.^2^^,^^3^ The principal rare morphologies reported, mucinous, ductal, signet ring cell, adenosquamous, sarcomatoid, and neuroendocrine, are recognized in standard classifications including American Joint Committee on Cancer (AJCC) reporting^6^ yet remain morphologic and are not incorporated into TNM staging. While not separately incorporated into TNM staging, invasive cribriform architecture is incorporated into Gleason pattern 4 scoring and thus into Grade Group assignment under the 2019 ISUP consensus^7^, and both cribriform growth and IDC-P are independently associated with biochemical recurrence and cancer-specific mortality in localized and locally advanced disease. Across subtypes, population-level data consistently show more advanced disease and worse outcomes than conventional acinar carcinoma.

Metastatic disease at presentation is highly enriched in NEPC (approximately 63%), sarcomatoid (33%), and adenosquamous (31%) tumors, intermediate in ductal and signet ring cell carcinoma (approximately 10%), and lowest in mucinous carcinoma.^2^^,^^3^ Ten-year overall survival (OS) mirrors this gradient; highest in mucinous (approximately 78%) and conventional acinar adenocarcinoma (approximately 71%), lowest in neuroendocrine (approximately 9%), sarcomatoid (approximately 15%), and adenosquamous (approximately 20%) disease, and intermediate in ductal and signet ring cell carcinoma (approximately 56%) (Figure 1). These values derive from older SEER and National Cancer Database cohorts predating contemporary therapy and indicate relative rather than current prognosis. Architectural patterns are independently prognostic, as invasive cribriform growth and IDC-P, distinguished in the 2019 ISUP consensus, predict recurrence, metastasis, and cancer-specific mortality, with IDC-P prevalence increasing from approximately 2% in low-risk disease to more than 50% in metastatic disease.^8^

**Figure 1.**
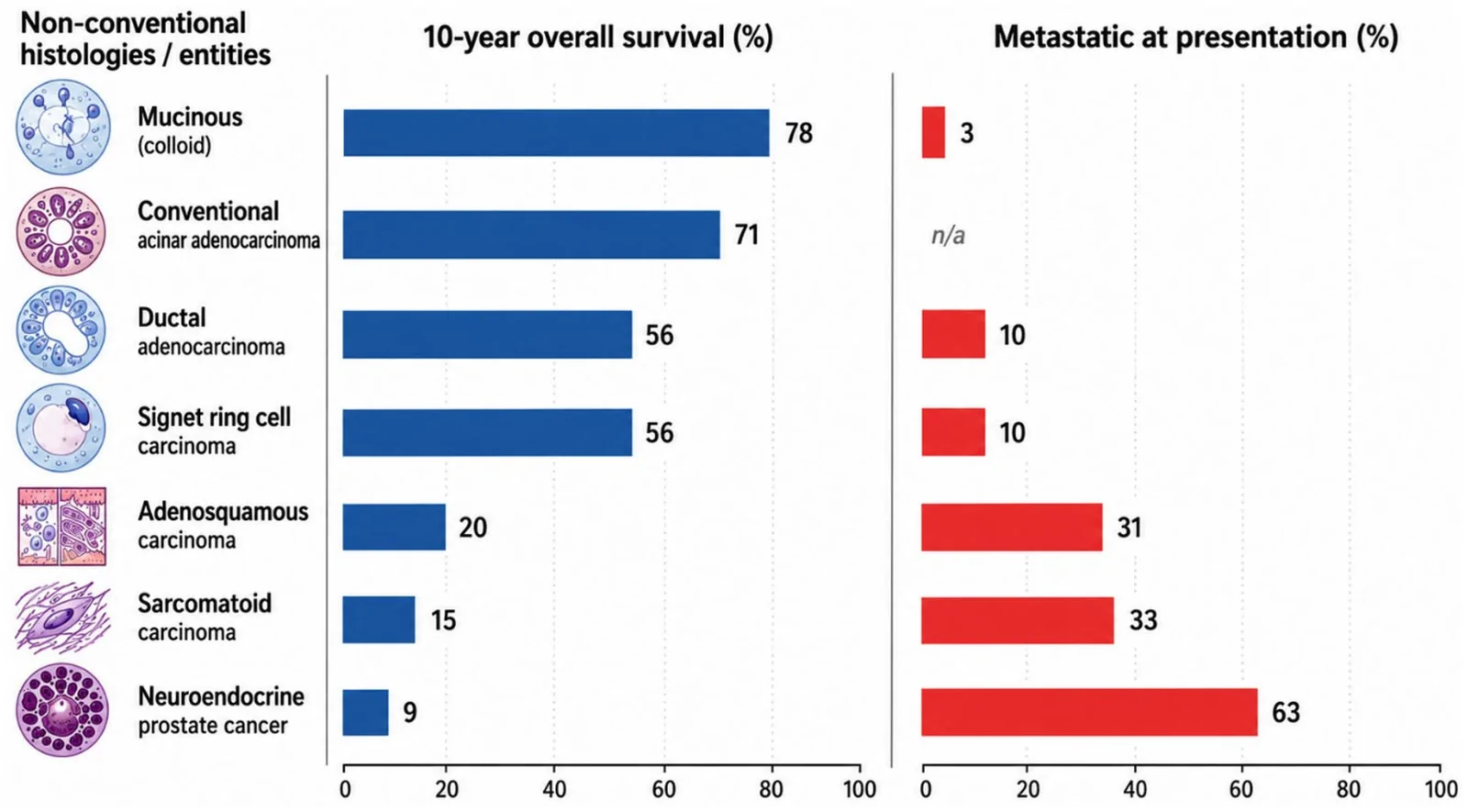
Outcomes across non-conventional histologies of prostate cancer. Ten-year overall survival (A) and frequency of metastatic disease at presentation (B). Approximate values from population-based datasets.^2^^,^^3^

## Diagnostic pathology and immunohistochemistry

Accurate diagnosis integrates morphology, clinical context, and selective immunohistochemistry, since classification directly governs the appropriateness of AR-directed therapy. The 2022 WHO classification separated subtypes, that has distinct morphology with implications related to prognosis and/or differential diagnosis, from unusual architectural patterns and formally recognized treatment-related neuroendocrine prostate cancer, reflecting therapy-driven lineage plasticity.^1^ Uncommon histologies remain under-recognized in routine practice: a multi-institutional assessment found that a comprehensive list of these entities was reported by pathologists in fewer than half of centers, underscoring the need for hypothesis-driven immunohistochemistry and expert genitourinary pathology review to avoid missed molecular-testing triggers.^9^

Immunohistochemistry should be hypothesis-driven to confirm prostatic origin, distinguish intraductal from invasive disease, identify neuroendocrine differentiation, exclude secondary tumors (Table 1)^10^ and is especially important in mucinous and signet ring morphologies, where gastrointestinal or urothelial malignancies may mimic prostatic tumors. GATA3 and CDX2 help exclude urothelial and gastrointestinal primaries, respectively, while NKX3.1, prostate-specific membrane antigen (PSMA), and ERG support prostatic origin; a panel is preferred because individual antigens may be lost in dedifferentiated disease. Treatment-emergent neuroendocrine differentiation should be suspected with rapid progression, visceral metastases, or PSA-discordant disease, and IDC-P or extensive cribriform architecture should trigger somatic and germline testing given their association with genomic instability and inherited DDR defects. Next-generation sequencing resolves ambiguous cases and identifies actionable alterations morphology cannot reveal; key associations are summarized in Table 2.

**Table 1.** Immunohistochemical markers in the diagnostic evaluation of non-conventional prostate carcinomas.

Category	Markers	Diagnostic utility
**Neuroendocrine differentiation**	Synaptophysin, chromogranin A, INSM1	Supports neuroendocrine phenotype^1^^,^^10^
**Luminal differentiation**	AR, PSA, NKX3.1	Indicates retained AR dependence
**Prostatic origin**	NKX3.1, PSA, PAP, ERG, PSMA	Confirms prostatic origin; helps distinguish IDC-P^7^
**Basal phenotype**	p63, CK5/6, CK34βE12	Confirms basal/adenoid cystic differentiation^11^
**Urothelial differentiation**	GATA3, Uroplakin, p63	Suggests urothelial origin
**Gastrointestinal differentiation**	CDX2, CK20, SATB2	Suggests gastrointestinal origin
**Emerging therapeutic target**	DLL3	Marker of neuroendocrine differentiation; identifies candidates for DLL3-directed therapy^12^^,^^13^

Abbreviations: AR, androgen receptor; IDC-P, intraductal carcinoma of the prostate; PAP, prostatic acid phosphatase; PSA, prostate-specific antigen; PSMA: prostate-specific membrane antigen.

**Table 2.** Key molecular alterations and therapeutic implications by histological subtype, entity, and adverse architectural pattern.

Histological subtype/pattern	Key molecular alterations	Associated biomarker (enrichment)	Therapeutic implication
**Neuroendocrine (NEPC)**	RB1 loss, TP53 mutation, ±PTEN loss; AURKA/MYCN; epigenetic reprogramming^4^^,^^14^	DLL3 expression	Platinum chemotherapy; DLL3-directed and other cell-surface engagers
**Aggressive-variant (AVPC)**	≥2 of TP53, RB1, PTEN^15^	Combined tumor-suppressor signature	Platinum-based chemo ± immunotherapy
**Cribriform/IDC-P**	BRCA2 and DDR deficiency; bi-allelic BRCA2 + PTEN loss (IDC-P); high CNA, c-MYC^16^^,^^17^^,^^18^	BRCA2/HRR status	PARP inhibitors;
**Ductal adenocarcinoma**	DNA-repair gene defects; MMR deficiency/MSI in a subset^5^^,^^19^^,^^20^	MMR/MSI status	PARP inhibitors; immune checkpoint blockade if dMMR/MSI-H
**Adenosquamous**	TP53, RB1, PTEN; occasional BRCA2/CDK12^21^^,^^22^	AVPC-like profile	Platinum-based chemotherapy; profiling
**Sarcomatoid**	TP53 loss, PTEN loss; EMT (ZEB1/SNAIL/TWIST); ERG in subset^23^	AVPC-like/EMT	Platinum chemotherapy; sarcoma-type regimens
**Adenoid cystic (basal)**	MYB::NFIB fusion; PTEN loss; EGFR^11^	MYB::NFIB	Surgery ± radiotherapy; AR-independent
**Signet ring cell**	Limited data; ERG in subset; AR-indifferent overlap^24^^,^^25^	NA	High-risk paradigms; profiling
**Mucinous (colloid)**	No distinct vulnerability^26^	NA	Standard risk-adapted management

Listed alterations and associations are approximate, synthesized from heterogeneous cohorts, registries, and case series, vary by assay and disease stage, and represent enrichments rather than deterministic rules; therapeutic implications are informed by current ESMO and NCCN guidelines.^27^^,^^28^

Abbreviations: AVPC, aggressive-variant prostate cancer; CNA, copy-number alterations; DDR, DNA damage repair; dMMR, mismatch-repair deficient; EMT, epithelial-to-mesenchymal transition; HRR, homologous recombination repair; IDC-P, intraductal carcinoma; MSI(-H), microsatellite instability (-high); NEPC, neuroendocrine prostate cancer.

## Non-conventional prostate cancer histologies as biological states: molecular determinants

### Neuroendocrine prostate cancer

NEPC is a biologically distinct and highly aggressive subset that arises through 2 partially overlapping evolutionary trajectories, namely a rare de novo small-cell/neuroendocrine carcinoma and a more common treatment-emergent form (t-NEPC) that develops under the selective pressure of AR-targeted therapy (Figure 2A). Identified in roughly 15%-20% of metastatic castration-resistant prostate cancer (mCRPC),^29^^,^^30^ it is a major mechanism of resistance and lineage adaptation. Clinically it shows rapid progression, visceral metastases, and PSA disproportionately low for tumor burden, features that should prompt suspicion of lineage transformation on AR-directed therapy. Mixed histology and intermetastatic heterogeneity are common,^31^ limiting archival tissue; circulating tumor DNA (ctDNA) can capture the transition and enable serial monitoring when tissue is inaccessible.^32^

**Figure 2.**
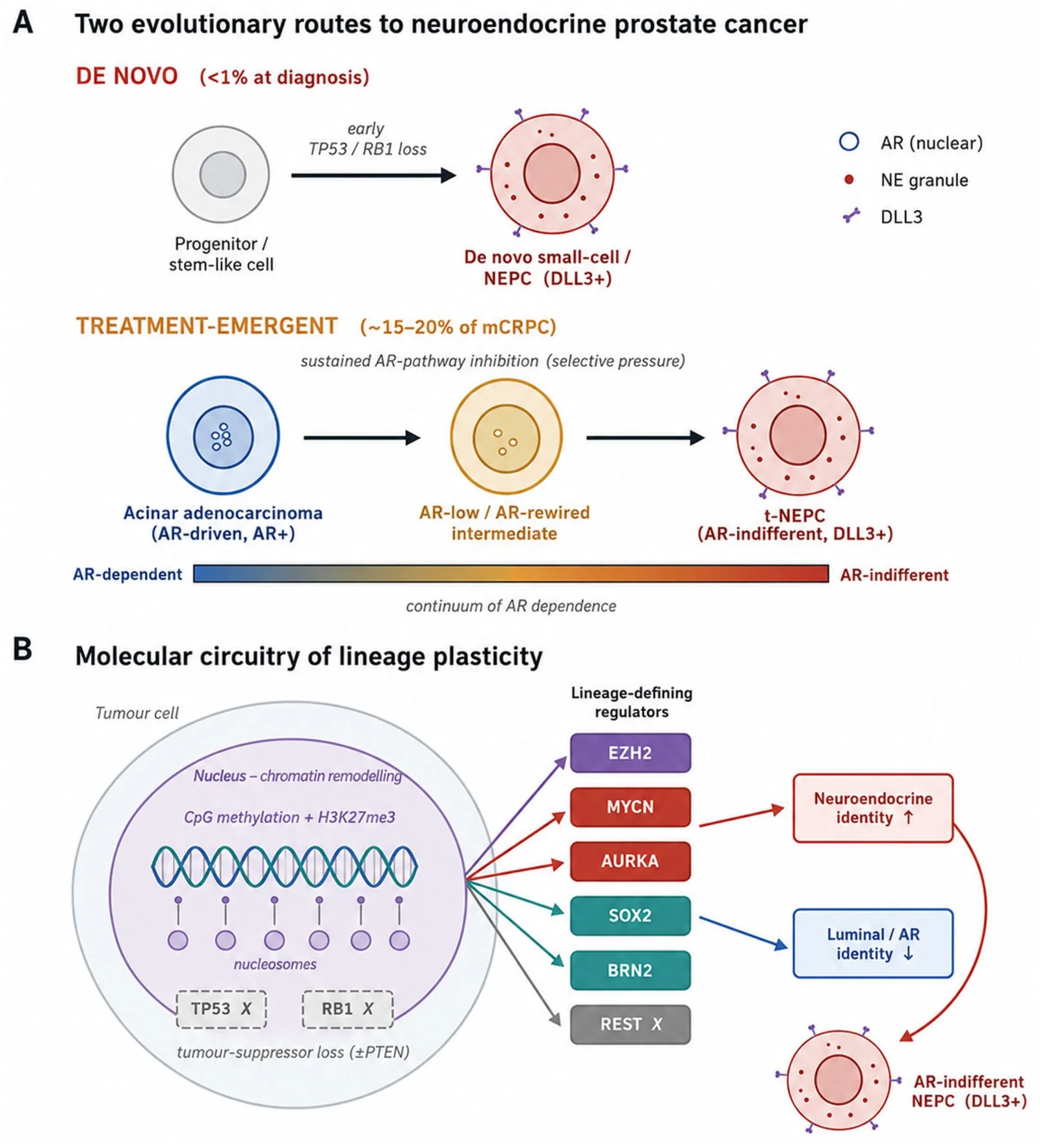
Biology of neuroendocrine differentiation. (A) Two evolutionary routes to neuroendocrine prostate cancer: rare de novo small-cell/neuroendocrine carcinoma and the more common treatment-emergent form (t-NEPC) arising from acinar adenocarcinoma under sustained AR-pathway inhibition, along a continuum of AR dependence. (B) Molecular circuitry of lineage plasticity: combined TP53/RB1 (±PTEN) loss enables epigenetic and transcriptional reprogramming (EZH2, MYCN, AURKA, SOX2, BRN2, REST loss) toward an AR-indifferent, DLL3-positive neuroendocrine phenotype. mCRPC, metastatic castration-resistant prostate cancer. The 2 routes also differ in prevalence: de novo NEPC is rare (under 1% of cases at presentation), whereas t-NEPC accounts for roughly 15%-20% of metastatic castration-resistant prostate cancer; separately, approximately 63% of patients diagnosed with NEPC overall (a figure dominated by the rarer de novo, more often metastatic-at-diagnosis presentations) have metastatic disease at diagnosis. These percentages use different denominators and are not contradictory.

### De novo vs treatment-emergent NEPC

De novo neuroendocrine carcinoma is rare, representing under 1% of cases at presentation; it usually arises without a documented history of adenocarcinoma, often shows pure small-cell or large-cell neuroendocrine morphology, and frequently carries early combined RB1 and TP53 loss. t-NEPC is clonally derived llfrom a preexisting adenocarcinoma, shares its founder alterations,^4^ and often presents as mixed, amphicrine, or AR-low intermediate phenotypes rather than pure small-cell disease;^33^ because it emerges late and heterogeneously, recognition depends on a high index of suspicion and repeat metastatic biopsy at atypical progression. Cheng et al.^34^ performing single-cell analyses, extended this further upstream. They demonstrated that rare, highly plastic neuroendocrine- and CRPC-like cells are already detectable within hormone-naïve primary tumors, well before any treatment pressure. Their subsequent expansion follows a bifurcated AR-dependent and AR-independent trajectory, and their presence in early disease correlates with recurrence and metastasis, reinforcing the rationale for identifying lineage-plastic biology before overt transformation.

Mechanistically, NEPC is driven by lineage plasticity coupled to genomic instability and epigenetic remodeling (Figure 2B). Paired-biopsy whole-exome and methylation analyses show that CRPC-adenocarcinoma and CRPC-NE share genomic overlap yet diverge epigenetically,^35^ supporting divergent clonal evolution rather than independent origin.^4^^,^^36^ Combined TP53 and RB1 loss, often with PTEN inactivation, is the central enabling event, sufficient to drive plasticity, metastasis, and antiandrogen resistance through derepression of reprogramming factors.^37^^,^^38^^,^^39^ The state is orchestrated by EZH2, MYCN, AURKA, SOX2, BRN2, and FOXA2,^14^ with loss of REST releasing neuronal networks.^40^ SOX2 promotes plasticity in TP53/RB1-deficient tumors.^38^ EZH2 is a central node in this circuitry, acting beyond canonical polycomb repression; its catalytic activity cooperates with aberrant DNA methylation to enforce the neuroendocrine program, and its deletion or pharmacological inhibition in murine and patient-derived NEPC models triggers genome-wide methylation rewiring, hypomethylation and upregulation of neuroendocrine-lineage genes with reciprocal hypermethylation and repression of polycomb targets and then positioning hypomethylated regions to accumulate the repressive mark H3K27me3.^41^ Because diverse neuroendocrine carcinomas converge on this shared epigenetic landscape, EZH2 and its crosstalk with DNA-methylation machinery provide a mechanistic rationale for epigenetic therapy.^41^^,^^42^

AR signaling is not uniformly extinguished but may be transcriptionally rewired, generating AR-low intermediate states that retain partial activity while acquiring neuroendocrine features; integrative analyses show lineage identity decoupled from morphology, with residual androgen signaling in tumors displaying neuroendocrine programs.^40^ Double-negative (AR-negative, neuroendocrine-negative) tumors sustained through FGF and MAPK signaling represent a further plastic state,^43^^,^^44^ supporting a continuum of AR dependence rather than a binary switch.

AVPC is a clinically defined syndrome broader than histologic NEPC, encompassing AR-indifferent, lineage-plastic states with aggressive behavior, early hormonal resistance, and enrichment for TP53, RB1, and PTEN alterations (Table 3).^15^^,^^29^ It is more common than once appreciated, with a substantial proportion of men progressing to CRPC meeting at least 1 AVPC criterion, and clinically or molecularly defined AVPC carries significantly shorter overall survival (hazard ratio 2.81; 95% CI 1.87-4.22) vs non-AVPC in a recent meta-analysis, with enrichment among high-volume, visceral, de novo metastatic, and tumor-suppressor-altered disease.^45^ A combined tumor-suppressor signature also predicts reduced benefit from AR-pathway inhibition in the hormone-sensitive setting.^46^ In practice the criteria are most useful as a prompt for metastatic biopsy and expanded testing; elevated neuron-specific enolase, chromogranin A, and CEA, though nonspecific, carry independent prognostic weight.^30^ These overlapping but non-interchangeable categories are worth distinguishing explicitly: histologic NEPC is defined by neuroendocrine morphology; clinically defined AVPC by the clinical/radiographic criteria in Table 3, independent of morphology or genomics; molecular AVPC by a combined tumor-suppressor signature (≥2 alterations among TP53, RB1, and PTEN); and double-negative (AR-negative, neuroendocrine-negative) disease by loss of both luminal and neuroendocrine programs. A tumor can meet clinical AVPC criteria without neuroendocrine morphology or a defined molecular signature, and double-negative disease can arise without meeting formal AVPC criteria.

**Table 3.** Clinical and molecular criteria defining aggressive-variant prostate cancer (AVPC).

Criterion	Clinical/molecular feature	Biological implication
**Small-cell/NE histology**	Pure or mixed small-cell/neuroendocrine morphology	Established AR-indifferent phenotype
**Visceral metastases**	Liver, lung, brain, or other visceral involvement	Lineage plasticity; poor prognosis
**Predominantly lytic bone metastases**	Osteolytic rather than osteoblastic lesions	Aggressive variant biology
**Bulky disease**	Bulky primary tumor or lymphadenopathy (≥5 cm)	Rapid growth; high disease burden
**Low PSA-high volume discordance**	Extensive metastatic burden despite low PSA	Reduced dependence on AR signaling
**Elevated neuroendocrine markers**	Chromogranin A or NSE, with elevated LDH or CEA	Supports neuroendocrine differentiation^30^
**Short response to ADT**	Progression within ≤6 months of ADT	Intrinsic hormonal resistance
**Rapid progression after hormonal therapy**	Early CRPC despite AR-targeted treatment	Treatment-emergent lineage plasticity
**AVPC molecular signature**	≥2 alterations among TP53, RB1, and PTEN	Molecular correlate of aggressive biology^15^^,^^47^

Abbreviations: ADT, androgen deprivation therapy; CEA, carcinoembryonic antigen; CRPC, castration-resistant prostate cancer; LDH, lactate dehydrogenase; NE, neuroendocrine; NSE, neuron-specific enolase; PSA, prostate-specific antigen.

NEPC spans classical small-cell carcinoma, mixed adenocarcinoma-neuroendocrine tumors, amphicrine phenotypes, and double-negative disease. Synaptophysin, chromogranin A, and INSM1 support diagnosis, though expression is heterogeneous and morphology remains paramount,^10^^,^^33^ and luminal markers (PSA, ERG, and NKX3.1) are often reduced but not absent. It carries the poorest prognosis among these histologies, with 10-year OS around 9%.^2^

At the cell surface, lineage transition reshapes the antigenic landscape in ways central to both diagnosis and later therapeutic targeting. The prototype is DLL3, an inhibitory Notch ligand aberrantly expressed in the majority of NEPC yet largely absent from benign prostate and conventional adenocarcinoma, making it a specific marker of neuroendocrine differentiation.^12^^,^^48^^,^^49^^,^^50^ Transition is frequently accompanied by loss of prostate-lineage antigens, notably PSMA, alongside gain or retention of alternative surface targets including STEAP1, KLK2, TROP-2, PSCA, and CEACAM5.^51^^,^^52^^,^^53^^,^^54^^,^^55^ This shifting surface phenotype underpins the targeted strategies discussed below, and DLL3 immunohistochemistry on metastatic biopsy is increasingly recommended whenever neuroendocrine transformation is confirmed or suspected.^12^

### Ductal adenocarcinoma, intraductal carcinoma, and cribriform architecture

Ductal adenocarcinoma (DAC) and cribriform architectural patterns (IDC-P, invasive cribriform growth in acinar adenocarcinoma) are best viewed as phenotypic manifestations of genomic instability rather than discrete entities and frequently coexist. DAC accounts for approximately 1%-2% of cases when mixed patterns are counted and far less in pure form^56^; it behaves aggressively, more often presenting with locally advanced (T3-T4) and metastatic disease, higher recurrence, and inferior survival, with a more than 4-fold increased risk of metastatic presentation and unusual sites such as peritoneum and penis. Population data suggest better outcomes with radical prostatectomy, though this is likely confounded by selection of fitter patients.^57^

One of the most consistent molecular associations is DDR deficiency. DAC, IDC-P, and cribriform architecture are enriched for BRCA2, ATM, and PALB2 alterations, often with complex rearrangements.^5^^,^^58^ BRCA2-deficient tumors form a distinct subset with early genomic instability, high copy-number burden, c-MYC amplification, and frequent ductal/intraductal morphology;^16^^,^^59^ patients with germline BRCA2 mutations show BRCA2-RB1 co-deletion and inferior survival.^18^ IDC-P has also been reported as an independent prognostic factor for shorter overall survival in men with metastatic prostate cancer treated with AR-pathway inhibitors, based on retrospective cohort evidence.^60^^,^^61^ The relationship between morphology and genotype is nuanced. Bi-allelic BRCA2 inactivation in combination with PTEN loss was associated with IDC-P, whereas germline BRCA2 status alone did not reliably predict its presence, supporting genomic rather than purely morphologic criteria for selecting patients for germline testing.^17^ ATM alterations confer more heterogeneous, less PARP-sensitive biology, and CDK12 inactivation defines a tandem-duplication subset with increased neoantigen load and investigational checkpoint sensitivity.^5^ Ductal differentiation is independently enriched for MMR deficiency with pathogenic MMR alterations found in roughly 40% of ductal-predominant tumors, mostly hypermutated with microsatellite instability^19^ and dMMR cancers reciprocally show ductal/intraductal histology and high grade.^20^ These findings have direct therapeutic implications. Randomized evidence supports PARP inhibition in HRR-deficient, particularly BRCA-altered mCRPC,^62^ and durable anti-PD-1 responses occur in the mismatch-repair-deficient or microsatellite-instability-high subset.^63^^,^^64^^,^^65^ Given that cribriform architecture in particular is common, universal testing is impractical; however, focal ductal or intraductal morphology, and cribriform disease presenting with high-risk or metastatic features, should prompt consideration of somatic and germline profiling.^66^^,^^67^

## Adenosquamous carcinoma

Adenosquamous carcinoma is an ultra-rare, highly aggressive tumor with mixed glandular and squamous differentiation, requiring exclusion of therapy-related change and secondary involvement; the squamous component is typically PSA- and prostatic acid phosphatase-negative and may express high-molecular-weight cytokeratin.^21^ It usually presents with advanced disease (metastases at diagnosis approximately 31%, 10-year survival approximately 20%)^2^ and often arises after androgen deprivation or radiotherapy, supporting therapy-induced reprogramming. Reported alterations in TP53, RB1, and PTEN with occasional BRCA2/CDK12 involvement indicate overlap with AVPC biology.^22^ Treatment is largely extrapolated from AVPC and squamous malignancies. Surgery is appropriate for localized disease, whereas platinum-based chemotherapy and comprehensive molecular profiling are recommended in the advanced setting.

## Sarcomatoid carcinoma

Sarcomatoid carcinoma is a rare biphasic epithelial-mesenchymal malignancy representing extreme lineage plasticity through epithelial-to-mesenchymal transition (EMT). Many cases follow prior adenocarcinoma years after treatment, supporting therapy-driven clonal evolution. Presentation is aggressive, with high metastatic propensity, low PSA relative to burden, and poor prognosis (metastases at diagnosis approximately one-third, 10-year survival approximately 15%).^2^^,^^23^ EMT is driven by ZEB1, SNAIL, and TWIST with TP53 and PTEN loss, while shared ERG rearrangements support common clonal origin with adenocarcinoma. Management follows aggressive-prostate-cancer and soft-tissue-sarcoma paradigms, commonly platinum-based, with genomic profiling recommended.

## Adenoid cystic (basal cell) carcinoma

Adenoid cystic (basal cell) carcinoma is an ultra-rare entity (approximately 100 reported cases) with basal cell differentiation, a distinct immunophenotype (p63, high-molecular-weight cytokeratins), and typically absent PSA. Historically considered indolent, it often presents with locally advanced disease and may behave aggressively. Its defining feature in a subset is the MYB::NFIB fusion, paralleling salivary adenoid cystic carcinoma;^11^ in the largest prostatic series it was detected by fluorescence in situ hybridization in roughly half of cases and was largely confined to the adenoid cystic pattern while absent from solid-pattern basal cell carcinomas and benign proliferations, supporting its value as a diagnostic discriminator.^68^^,^^69^ The fusion drives constitutive MYB activation, and PI3K/AKT and NOTCH involvement with PTEN loss and EGFR overexpression has been reported, though none is yet validated as prognostic or predictive and no fusion-directed therapy exists. These non-luminal, AR-independent tumors respond poorly to androgen deprivation; radical surgery with negative margins, often with radiotherapy, is the cornerstone, while systemic options remain poorly defined.

## Primary prostatic signet ring cell carcinoma

Primary signet ring cell carcinoma (SRCC) is extremely rare and highly aggressive (fewer than 200 reported cases),^70^ often following prior adenocarcinoma years later, supporting clonal dedifferentiation.^24^^,^^25^ Defined by intracytoplasmic mucin vacuoles displacing the nucleus, it presents with advanced disease, low PSA relative to burden, and poor prognosis (metastases at diagnosis approximately 10%, 10-year survival approximately 56%).^2^ Diagnosis relies primarily on exclusion because primary prostatic SRCC is far less common than its gastrointestinal or bladder counterparts. An organ-specific immunohistochemical panel is therefore essential, with NKX3.1, PSA, and prostatic acid phosphatase supporting a prostatic origin, whereas CDX2, TTF-1, and CK20 favor a secondary tumor. Prostatic SRCC has not been comprehensively sequenced; its biology is extrapolated from SRCC of other sites, where E-cadherin loss through CDH1 alteration is a recurrent, morphology-defining event with frequent TP53 and PIK3CA mutations, though whether prostatic SRCC shares this CDH1-centred biology is unknown.^71^^,^^72^ Available prostatic data suggest overlap with AR-indifferent disease and ERG rearrangements in a subset. No histology-specific standard exists; management follows high-risk paradigms with profiling for DNA-repair and mismatch-repair defects.

## Mucinous adenocarcinoma and secondary tumors

Mucinous adenocarcinoma is defined by at least 25% extracellular mucin and includes colloid adenocarcinoma, adenocarcinoma with mucinous features, and rare mucin-producing urothelial-type tumors; secondary colorectal or urothelial tumors are important mimics that must be excluded.^26^ Unlike histologic subtype, mucinous adenocarcinoma does not appear to confer a distinct molecular vulnerability or adverse prognosis. This is consistent with molecular data showing that colloid prostatic adenocarcinoma expresses ERG, a surrogate for TMPRSS2::ERG fusion, at frequencies comparable to conventional acinar adenocarcinoma, supporting its classification as an unusual histological pattern of conventional adenocarcinoma rather than as a distinct biological entity.^73^ Institutional series report outcomes comparable to or better than acinar adenocarcinoma after radical treatment, with the lowest metastatic presentation and 10-year survival approaching 78%,^2^ contingent on excluding a mucin-producing secondary tumor. Management otherwise follows standard risk-adapted care.

## Therapeutic framework

In advanced disease, the pivotal question is whether tumors remain AR-driven or have transitioned toward AR-indifferent biology (Figure 3). AR-directed therapy suits preserved luminal differentiation and PSA-concordant progression; conversely, rapid progression on AR inhibition, visceral metastases, bulky disease with low PSA, lytic bone lesions, elevated LDH or CEA, neuroendocrine morphology, or combined TP53/RB1/PTEN loss should prompt consideration of AVPC or NEPC. Metastatic biopsy is critical, since mixed histologies are common and treatment should reflect the dominant phenotype; consistent with ESMO and NCCN guidance, morphology is a trigger for molecular testing, not a treatment biomarker in itself.^27^^,^^28^ Within this framework, guideline-supported approaches with an established evidentiary basis–platinum-based chemotherapy in small-cell/neuroendocrine disease, PARP inhibition in appropriately selected HRR-deficient disease, and checkpoint blockade in dMMR/MSI-H tumors–should be distinguished from investigational, target-directed strategies (DLL3, STEAP1, KLK2, EZH2, and alternative radioligands) discussed below, which currently lack regulatory approval and are supported chiefly by early-phase or single-arm data.

**Figure 3.**
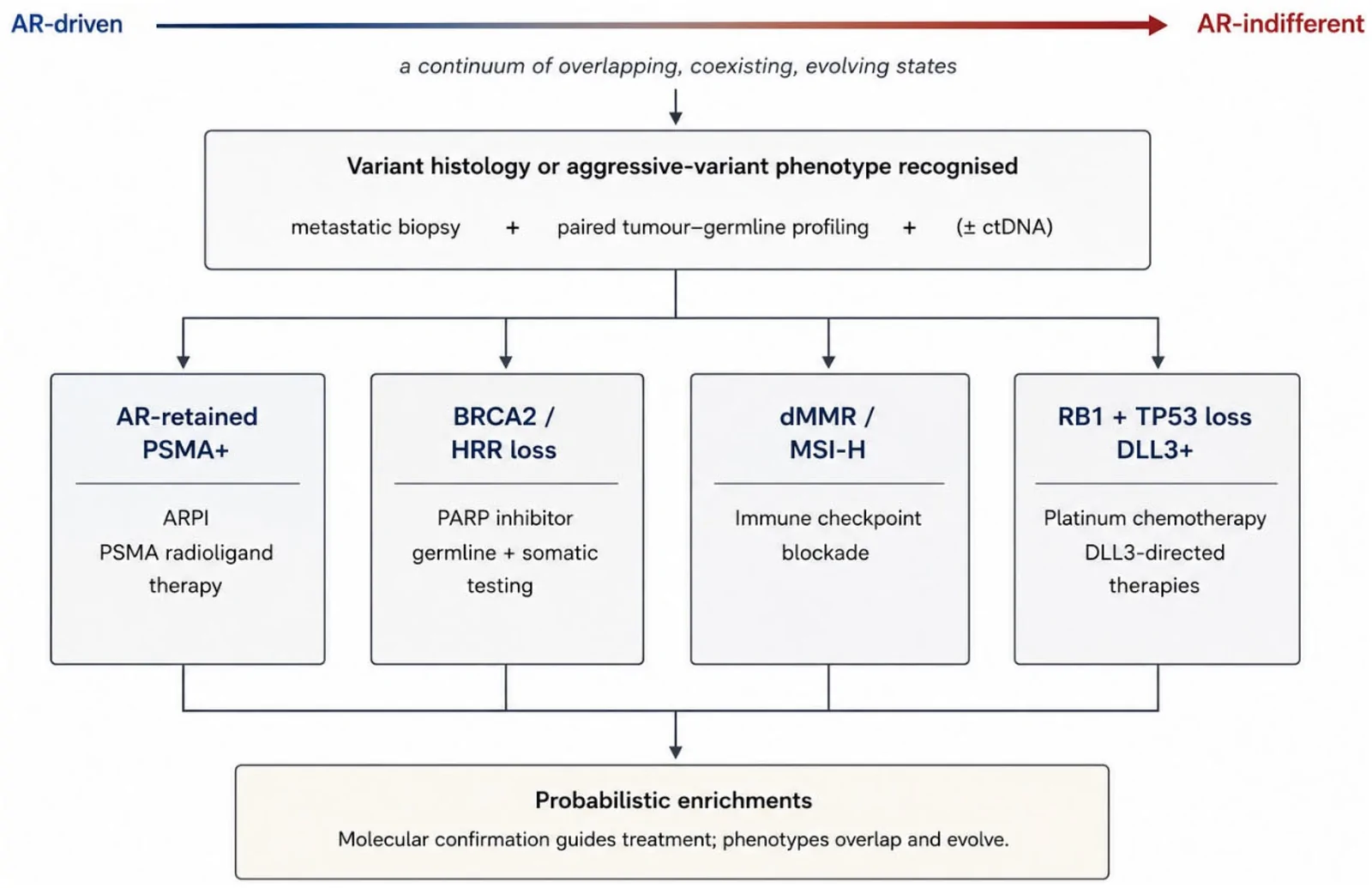
A biology-driven therapeutic framework. Non-conventional histology or an aggressive-variant phenotype should prompt metastatic biopsy and paired tumor-germline molecular profiling (±ctDNA); the molecular result, not morphology alone, then informs therapy along a continuum of AR dependence. The associations shown are probabilistic enrichments rather than deterministic rules, and phenotypes overlap, coexist, and evolve, so mixed and discordant results are common. The framework’s specific contribution is the entry point–recognition of non-conventional histology or an aggressive-variant phenotype triggering biopsy and paired molecular profiling–rather than the treatment options themselves, which follow established biomarker-selected principles once the molecular subgroup is confirmed. ARPI, AR-pathway inhibitor; ctDNA, circulating tumor DNA; dMMR, mismatch-repair deficient; HRR, homologous recombination repair; MSI-H, microsatellite instability-high; PSMA, prostate-specific membrane antigen.

Platinum-based chemotherapy is the backbone for small-cell and neuroendocrine disease, by extrapolation from small-cell lung cancer (SCLC).^74^ Regimen choice is guided by phenotype; platinum-etoposide mirrors small-cell lung cancer protocols and is generally preferred for pure small-cell or classical neuroendocrine morphology,^75^ whereas platinum-taxane combinations are commonly used in mixed or aggressive-variant disease that retains an adenocarcinoma component, covering both compartments. A contemporary meta-analysis confirms that platinum regimens outperform non-platinum chemotherapy in aggressive-variant and treatment-related neuroendocrine disease,^45^^,^^47^ with higher objective response rates (46% vs 19%) and, in clinically and morphologically defined AVPC, longer progression-free and overall survival; benefit is less certain, and responses more often partial, in aggressive-variant disease lacking overt small-cell morphology. A Phase I/II randomized trial enrolling heavily pretreated mCRPC patients showed improved activity of carboplatin plus cabazitaxel vs cabazitaxel alone in terms of progression-free survival, with 4.5 months (95% CI; 3.5-5.7) in the cabazitaxel group versus 7.3 months (5.5-8.2) in the combination group (HR = 0.69, 95% CI; 0.50-0.95, *P* = .018) in the intention-to-treat population, with greatest benefit in aggressive-variant features.^76^ Re-treatment with platinum may be considered at later progression depending on the prior treatment-free interval, and fitness, organ function, and cumulative toxicity should guide regimen selection given the hematologic burden of these combinations. Later-line options extrapolated from SCLC include lurbinectedin and topotecan.^77^

Immunotherapy-based combinations may partially overcome the immune exclusion of lineage-plastic disease. The phase 2, single-arm CHAMP trial^78^ of cabazitaxel-carboplatin with nivolumab and ipilimumab in AVPC or NEPC, reported in abstract form, met its primary endpoint, with a 6-month immune-modified radiographic progression-free survival rate of 74% (28 of 38 evaluable patients; 90% CI; 63-100), exceeding the historical benchmark for chemotherapy alone (55%; *P* = .013); median immune-modified radiographic progression-free survival was 12 months (95% CI; 9.1-14), the objective response rate by immune-modified criteria was 37%, and median overall survival was also 12 months, within the historical range reported for cabazitaxel plus carboplatin alone in AVPC (9.9-17.4 months); notably, 23% of patients were alive at 2 years, indicating durable benefit in a subset despite frequent RECIST progression.

In the phase 2 C3NIRA study, reported in abstract form, the addition of cetrelimab (anti-PD-1) to cabazitaxel-carboplatin followed by niraparib maintenance improved both PFS and OS in men with AVPC.^79^

Lineage-directed and biomarker-driven strategies are advancing rapidly, exploiting the AR-independent surface and transcriptional program of neuroendocrine disease. DLL3 is the prototypical target; in the phase 1 b DeLLpro-300 study, which enrolled 40 patients with NEPC, the DLL3-directed bispecific T-cell engager tarlatamab achieved objective responses of 10.5% (95% CI; 2.9-24.8) overall and 22.2% (95% CI; 6.4-47.6) in DLL3-positive tumors with none in DLL3-negative or DLL3-unknown disease, with a median response duration of 7.3 months and a manageable, cytokine-release-predominant profile.^80^^,^^81^^,^^82^

The DLL3 pipeline is expanding with additional bispecific and trispecific engagers, such as gocatamig, obrixtamig, peluntamig, ZG006, and DLL3-directed radioligand and imaging agents,^13^^,^^83^ while other engagers extend surface targeting across AR-independent disease.

The STEAP1-directed xaluritamig achieved PSA50 responses in roughly half of heavily pretreated patients,^84^ and the KLK2-directed pasritamig showed activity with a favorable safety profile.^85^ Transcriptional dependencies are also emerging as therapeutic vulnerabilities. The functional cooperation between MYCN and Aurora kinase A, a key driver of the neuroendocrine program, provided the rationale for evaluating the Aurora kinase A inhibitor alisertib in a phase II trial that enrolled 60 men with castration-resistant or neuroendocrine prostate cancer; although the primary endpoint was not met (6-month radiographic progression-free survival 13.4%, median overall survival 9.5 months), exceptional and durable responses occurred in patients whose tumors showed Aurora-A and N-myc activation, illustrating both the promise and the limits of biomarker-selected targeting.^86^ EZH2 inhibition with AR-targeted therapy is likewise being pursued in molecularly defined NEPC, supported by the epigenetic crosstalk described above. In parallel, homologous recombination repair (HRR) defects, particularly BRCA2, support PARP inhibitors and MMR deficiency identifies checkpoint-blockade candidates,^62^^,^^63^ favoring early paired tumor-germline profiling once a non-conventional histology or aggressive-variant phenotype is recognized.

Molecular imaging should increasingly inform selection. Progressive PSMA loss accompanies transition toward AR-indifferent and neuroendocrine phenotypes, potentially limiting PSMA radioligand therapy, whereas DLL3, somatostatin receptor 2 (SSTR2), gastrin-releasing peptide receptor (GRPR), PSCA, and KLK2 are under investigation and may enable adaptive radiotheranostics.^55^^,^^87^

## Future perspectives and ongoing clinical trials

Development is shifting decisively toward biology-driven, biomarker-selected treatment, particularly in NEPC and AVPC. Chemoimmunotherapy and maintenance strategies combining PARP inhibition with immunotherapy have shown encouraging activity. At the same time, lineage-directed therapy has emerged as the most rapidly evolving field, with DLL3 leading the way through the development of bispecific and trispecific T-cell engagers, antibody-drug conjugates, and radioligands.^80^^,^^83^^,^^88^ Additional targets, including STEAP1, KLK2, and TROP-2, are further expanding the spectrum of surface-directed therapies, although most remain investigational (Table 4).^52^^,^^84^^,^^85^ Future trials should prioritize biomarker-driven designs integrating tissue genomics, ctDNA, circulating tumor cells, molecular imaging, and longitudinal sampling to detect lineage transitions and enable adaptive strategies. Because these histologies are individually rare, multi-institutional and basket designs, centralized pathology review, and equitable access to comprehensive profiling and expert genitourinary pathology will be essential to generate practice-changing evidence.

**Table 4.** Selected ongoing clinical trials in neuroendocrine and aggressive-variant prostate cancer (through ASCO 2026).

Trial (NCT)	Phase	Population	Strategy	Investigational agent
**CHAMP (NCT04709276)**	II	AVPC/NEPC	Platinum chemoimmunotherapy	Nivolumab + ipilimumab + carboplatin + cabazitaxel^78^
**PEAPOD-FOS (NCT05563558)**	II	AVPC mCRPC	Chemoimmunotherapy	Pembrolizumab + carboplatin + cabazitaxel
**C3NIRA (NCT04592237)**	II	AVPC mCRPC	PARP inhibition + IO maintenance	Niraparib + cetrelimab^79^
**DeLLpro-300 (NCT04702737)**	Ib	De novo/t-NEPC	DLL3-directed BiTE	Tarlatamab^80^
**MK-6070-001 (NCT04471727)**	I/II	DLL3+ NE cancers	DLL3-directed TriTE	Gocatamig (MK-6070/HPN328) ± atezolizumab^83^
**SKYBRIDGE (NCT05652686)**	I	DLL3+ NE tumors	DLL3/CD47 bispecific	Peluntamig (PT217)
**DAREON-5 (NCT05882058)**	II	DLL3+ SCLC/NEPC	DLL3/CD3 TCE	Obrixtamig (BI 764532)
**NCT07024277**	II	Metastatic NEPC	DLL3/CD3 trispecific	ZG006
**AMG 509 (NCT04221542)**	I	mCRPC	STEAP1 × CD3 TCE	Xaluritamig^84^
**NCT04898634**	I	mCRPC	KLK2 × CD3 TCE	Pasritamig^85^
**NCT06941480**	I	NEPC/NEC	DLL3-targeted radioligand	[177Lu]Lu-DTPA-SC16.56^13^

Abbreviations: AVPC, aggressive-variant prostate cancer; BiTE, bispecific T-cell engager; mCRPC, metastatic castration-resistant prostate cancer; NE, neuroendocrine; NEC, neuroendocrine carcinoma; NEPC, neuroendocrine prostate cancer; SCLC, small-cell lung cancer; TCE, T-cell engager; t-NEPC, treatment-emergent NEPC; TriTE, trispecific T-cell engager.

## Limitations and challenges

Several limitations apply. This is a narrative rather than systematic review, and much evidence derives from small retrospective series and case reports with selection and publication bias. Molecular characterization is uneven, most mature for neuroendocrine, ductal, intraductal, and cribriform disease and sparse for adenoid cystic, signet ring cell, and mucinous carcinomas, so the surrogate associations are strongest for the former. Many encouraging signals come from small early-phase trials or non-peer-reviewed abstracts, and platinum regimens produce only modest, short-lived responses (objective response rates roughly 20%-30%, progression-free survival 4-6 months^45^), further attenuated in pure small-cell, high-LDH, and bulky disease. Platinum sensitivity may partly reflect co-occurring DNA-repair defects rather than neuroendocrine differentiation, and combined TP53/RB1/PTEN loss has not consistently predicted benefit.^15^^,^^89^ T-cell engagers carry cytokine-release toxicity,^82^ and DLL3-directed strategies depend on standardized target assessment not yet uniformly available.^12^^,^^13^ The values presented are best read as approximate and hypothesis-generating, and the framework as decision support prompting molecular confirmation, not a deterministic rule; prospective, biomarker-driven validation is required.

## Conclusions

Rare histological morphologies of prostate cancer form a biologically distinct spectrum defined by lineage plasticity, genomic instability, therapeutic resistance, and adverse outcomes. Rather than representing fixed morphologic categories, many of these histologies reflect dynamic biological states shaped by clonal evolution and therapeutic pressure. Morphology therefore serves as an accessible, albeit imperfect, surrogate for therapeutically relevant molecular alterations and should prompt comprehensive molecular characterization rather than replace it. The principal examples are neuroendocrine differentiation as a surrogate for DLL3 expression with combined RB1 and TP53 loss; cribriform and intraductal architecture for BRCA2 and DNA-repair deficiency; and ductal differentiation for mismatch-repair deficiency in a subset.^90^ Framed this way, the pathology report becomes not an endpoint but a trigger for a defined set of molecular questions, each linked to a therapeutic class and to germline implications with the principal contribution of extending this surrogate logic across the full spectrum of rare histologies. Important caveats remain, including the probabilistic nature of morphology–genotype relationships, which should prompt rather than replace molecular testing, and the fact that the biological basis of platinum sensitivity in aggressive-variant disease remains uncertain and its clinical and genomic definitions do not always coincide; and lineage identity is increasingly decoupled from morphology, complicating any binary AR-dependent versus AR-indifferent classification. Reconciling these definitions and validating morphology-to-biology associations prospectively are the necessary next steps. Integrating expert histopathology with longitudinal molecular characterization, genomics, liquid biopsy, and molecular imaging, offers a practical bridge from the pathology report to biology-driven care.

## Data Availability

Data sharing is not applicable to this article, as no new datasets were generated or analyzed during this review.
